# Super-enhancers: novel target for pancreatic ductal adenocarcinoma

**DOI:** 10.18632/oncotarget.26704

**Published:** 2019-02-22

**Authors:** Chandrayee Ghosh, Santanu Paul, Prasad Dandawate, Sumedha S. Gunewardena, Dharmalingam Subramaniam, Cameron West, Shrikant Anant, Animesh Dhar

**Affiliations:** ^1^ Department of Cancer Biology, University of Kansas Medical Center, Kansas City, KS-66160, USA; ^2^ Department of Molecular and Integrative Physiology, University of Kansas Medical Center, Kansas City, KS-66160, USA; ^3^ Genzada Pharmaceuticals, Sterling, KS-67579, USA

**Keywords:** PDAC, super-enhancers, therapeutics, ChIP-Seq, cancer stem cells

## Abstract

Super-enhancers (SEs) are unique areas of the genome which drive high-level of transcription and play a pivotal role in the cell physiology. Previous studies have established several important genes in cancer as SE-driven oncogenes. It is likely that oncogenes may hack the resident tissue regenerative program and interfere with SE-driven repair networks, leading to the specific pancreatic ductal adenocarcinoma (PDAC) phenotype. Here, we used ChIP-Seq to identify the presence of SE in PDAC cell lines. Differential H3K27AC marks were identified at enhancer regions of genes including c-MYC, MED1, OCT-4, NANOG, and SOX2 that can act as SE in non-cancerous, cancerous and metastatic PDAC cell lines. GZ17-6.02 affects acetylation of the genes, reduces transcription of major transcription factors, sonic hedgehog pathway proteins, and stem cell markers. In accordance with the decrease in Oct-4 expression, ChIP-Seq revealed a significant decrease in the occupancy of OCT-4 in the entire genome after GZ17-6.02 treatment suggesting the possible inhibitory effect of GZ17-6.02 on PDAC. Hence, SE genes are associated with PDAC and targeting their regulation with GZ17-6.02 offers a novel approach for treatment.

## INTRODUCTION

Pancreatic ductal adenocarcinoma (PDAC) is a lethal malignancy and is the fourth leading cause of cancer-related mortality in the USA, due to its susceptibility to metastasis. Only one-fifth of Americans diagnosed with PDAC survive for a full year and the five-year survival rate is found to be <6% due to poor response to the currently approved therapeutics and increasing incidence of drug resistance. In 2019, an estimated 45,750 Americans (23,800 men and 21,950 women) will die of the disease [1]. By 2030, the disease is predicted to be the second leading cause of cancer-related deaths [2]. Although cancer research has advanced in a discerning novel and superior methods of diagnosis and therapies in molecular pathogenesis, PDAC remains a major unresolved health concern worldwide [3–5]. Lack of markers for early detection and screening programs even in high-risk populations, along with rapid invasion, metastasis, recurrence after surgery and developing resistance to standard therapies make PDAC a deadly disease [6]. The major reasons are substantial molecular heterogeneity of this tumor type and limited understanding of the molecular mechanism of PDAC progression. The key driver mutations and core signaling pathways that have been identified through genome sequencing efforts are not yet readily druggable. Recent investigations by Roe *et al.* [7] revealed how alteration in the transcription and enhancer landscape takes place during discrete stages of disease progression in PDAC mouse model. Thus, identifying novel therapeutic agents targeting enhancers related to disease progression is an imperative need for cancer research.

Hnisz *et al.* [8] defined super-enhancers (SEs) as large clusters of transcriptional enhancers that drive the expression of genes that outline cell identity. Epigenetic modifications such as DNA methylation [9] and histone modification have shown to regulate enhancers [9, 10]. Co-localization of murine embryonic stem cell (ESC) genomic sites by the master transcription factors OCT4, SOX2, and NANOG was initially highly predictive of enhancer activity [9]. However, in this study, they have produced an array of SEs in a wide range of human cell types and found that SEs are associated with genes that govern and define the biology of these cells. The most interesting finding was that the disease-associated variation is specifically enriched in the SEs of the disease-relevant cell types. This also encompasses that SEs are generated at oncogenes and other genes important in tumor pathogenesis. Later SEs were also defined as large regulatory units which could play a vital role in sustaining cancer cell identity and promoting addictive oncogenic transcription. Epigenetic modifications such as DNA methylation [10] and histone modification have been shown to regulate enhancers [10, 11]. Present technologies could give an insight into how enhancer activity and epigenetic changes at enhancer regions are related. Active and inactive enhancers, based on histone modifications such as H3K4me1 and H3K27ac could be distinguished [11]. As existing defects in cell-signaling pathways allow cancer cells to alter their normal programs of proliferation, transcription, growth, migration, differentiation, and death, hence reports suggest such reliance on SE-driven transcription for proliferation and survival offers a potent therapeutic mark for the targeting of cancer cells. Inhibition of the cellular machinery required for the assembly and maintenance of SEs might reduce oncogenic transcription and inhibit tumor growth [12]. Evan *et al*. stated that the driver oncogenic mutations do not specify the phenotype for different cancers; rather they all hack into the resident preconfigured super enhancer regenerative program of the target tissue and is probable in PDAC [13]. That observation suggested that the development of tumorigenesis is dependent on SEs transcription activity and could be a target for novel therapies [13].

Among many pathways of cancer progression that PDAC relies on, anomalous activation of the sonic hedgehog (SHH) pathway has shown in a variety of human cancers, including, basal cell carcinoma, malignant gliomas, medulloblastoma, leukemias, and cancers of the breast, lung, pancreas, and prostate [14]. The hedgehog (HH) signaling pathway is critical for the embryologic development of the pancreas thus aberrant HH signaling stimulates pancreatic carcinogenesis, stromal growth, and preservation of the tumor microenvironment. The canonical HH-pathway in the PDAC stroma has been targeted widely but has not yet lead to promising clinical results [15]. Thus, novel anticancer agents are still to be developed to target potential proteins acting in these pathways. The combined use of these inhibitors with standard anticancer treatments could allow researchers to attack tumors on many fronts.

In 2002, the World Health Organization (WHO) estimated that 80% of the world's population in developing countries depends on plants and traditional medicine practitioners to meet their primary health care needs [16]. Recent reports suggest that they can target multiple pathways and several cell types including cancer stem cells [17–19]. As a result, in the last decade, they have been investigated as potent anti-cancer agents. *Arum palaestinum* Boiss is an indigenous plant from the Middle East that is consumed as an herbal therapy against cancer [20]. Numerous cancer patients ingesting the plant had a beneficial effect thus a probable remedial proposition was foreseen after the active ingredients were identified. Cole *et al*. [20] have shown that fortified *Arum palaestinum* Boiss caused a reduction in live cells within prostate cancer spheroids and blocked tumor growth in mice without signs of toxicity. Isovanillin, linolenic acid, and β-sitosterol were identified to be the active ingredients contributing to anti-cancer activity. Later known quantities of these three chemical components were fortified ensuing in a compound designated as “GZ17”. However, in the present study, a new formulation of the compound was used in combination with other anti-cancer agents, harmine and curcumin resulting in a potent mixture (77% Isovanillin, 13% Harmine and 10% Curcumin) termed as “GZ17-6.02”. Harmine and curcumin are reported to be promising drug candidates for cancer therapy [21, 22] and the rationale behind formulating this mixture was to increase the efficacy of these agents and masking their probable toxicity on normal cells. In this study, we demonstrated that the SE landscape is significantly different in cancer vs non-cancer cells and GZ17-6.02 could reduce the H3K27ac of major master transcription factor genes with promising anticancer activity in both *in vivo* and *in vitro* system and could be novel therapeutics for PDAC.

## RESULTS

### SEs are associated with key identity genes in PDAC cells

**Figure 1:**
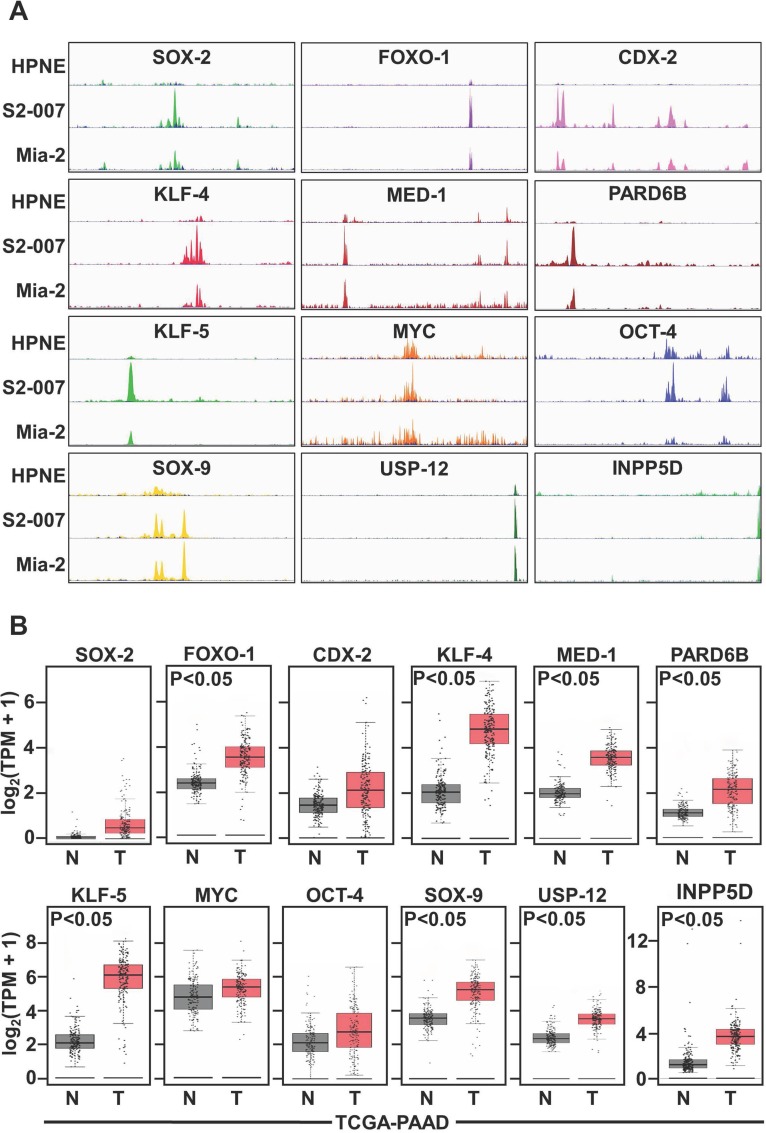

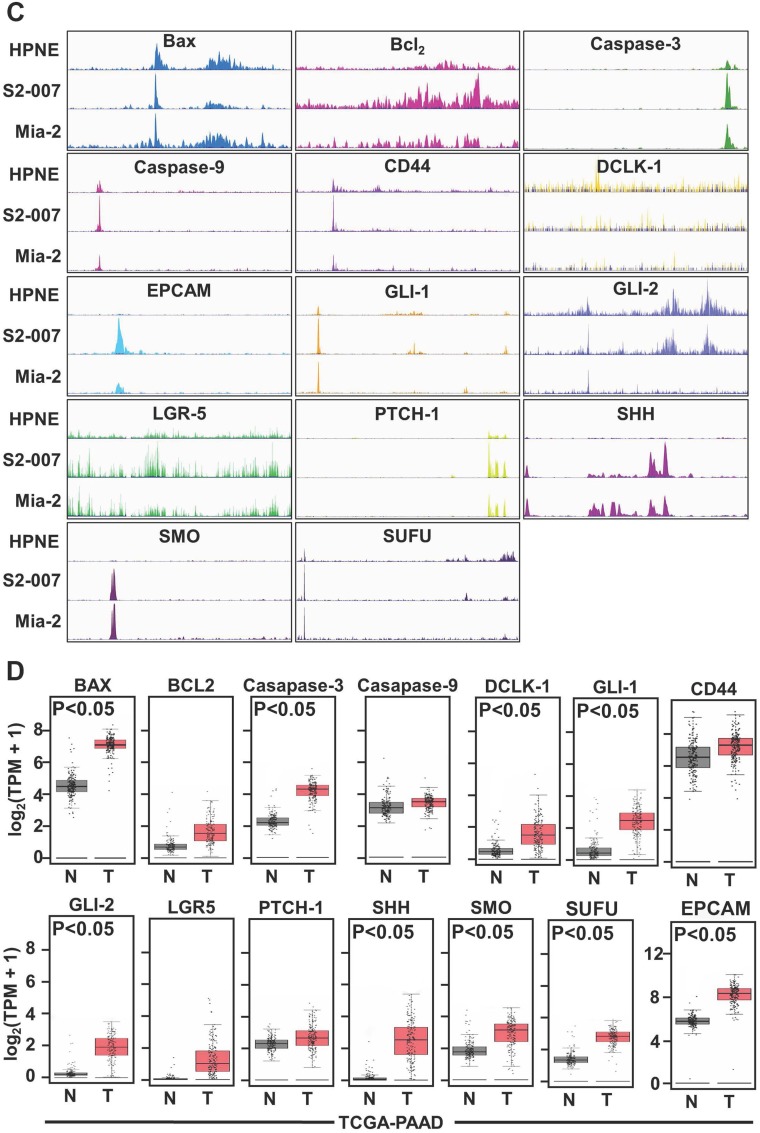
Binding activity of SEs in non-cancerous (HPNE) and in PDAC cells (MiaPaCa-2 and S2-007) using histone mark H3K27ac PDAC cells demonstrated significant binding of different enhancers in PDAC cells than non-cancerous cells, (**A**) Binding with SOX2, FOXO-1, CDX-2, KLF-4, MED-1, PARD6B, KLF-7, MYC, OCT-4, SOX-9, USP12, INPP5D and (**C**) with SHH pathway using ChIP-Seq with the H3K27me3 antibody. (**B** and **D**) TCGA data also suggested a higher expression of those genes in PDAC compared with normal tissue.

It has been reported previously that to activate the transcription, enhancers tend to associate with the adjacent genes. However, the gene type varied with cells emphasizing the role of SEs in cell identity. To assess whether the enhancer landscape becomes altered during PDAC progression and if so for which genes, we profiled genome-wide enrichment of H3K27ac in two cancer cells and one non-cancerous cell line. We generated high-quality ChIP-Seq dataset for H3K27ac in human PDAC cells lines (S2-007 and MiaPaCa-2) and one non-cancerous ductal cell line (HPNE) and examined the genome-wide occupancy for H3K27ac that was similar across the three samples except in few regions. We then examined the cancer-related genes associated with this SE domain. As predicted, it was observed that (Figure 1A) H3K27ac signal increased at *GAIN* regions [7] of the transcription factor genes' locus that are reported to upregulate transcription of oncogenes in the cancer cells. SOX2, FOXO-1, CDX-2, KLF-4, MED-1, PARD6B, KLF-5, MYC, OCT-4, SOX-9, USP-12, and INPP5D are all been reported to contribute in transcriptional upregulation of oncogenes thus playing a crucial role in imparting the oncogenic property corresponds with our observation. More than 90% of the H3K27ac regions lie outside of promoter regions and can be correlated with the expression changes of nearby genes, suggesting that they represent enhancer elements. However, we wanted to inspect the role and aberration of these genes in the patient sample database to confirm the findings. We found significant upregulation in mRNA expression for the above-mentioned genes in PDAC patients compared to normal (Figure 1B) from TCGA database. Further, we assessed the H3K27ac level associated with other genes related to apoptosis, stem cell markers, and SHH pathway. As mentioned, the SHH pathway is essential in the development of cancer. We found significantly higher acetylation peaks in the PDAC cell lines compared to normal (Figure 1C) confirming their upregulation in cancer. Information from the TCGA database supports our data most of the patient sample showed higher mRNA level of these genes (Figure 1D).

### mRNA transcription levels of SEs, stem cell markers and SHH could be targeted by GZ17-6.02

**Figure 2:**
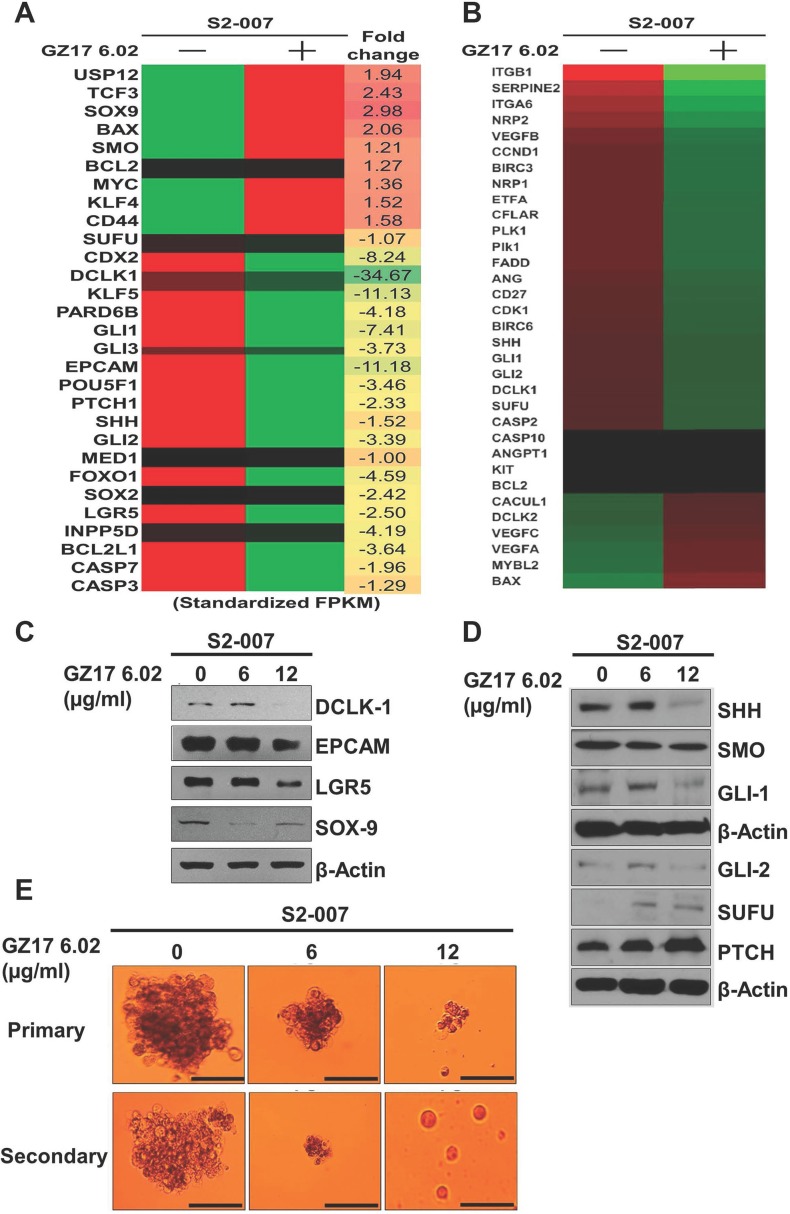
Detection of SEs related gene expression using heat map of RNAseq (**A** and **B**) following treatment with GZ17-6.02, S2-007 cells demonstrated impairment of expressions SEs related genes including pluripotent stem cell and cancer stem cell markers after treatment. (**C**) The CSC markers, (**D**) SHH pathway and (**E**) pancosphere formation significantly affected by GZ17-6.02 treatment.

To target the SE associated gene expression we used a novel mixture of natural compounds (phytochemicals) due to its antitumor properties. We performed some initial experiments with this mixture on the cell lines and found that it has prominent anticancer activity on the cancer cells. PDAC cells, S2-007 and MiaPaCa-2 were treated with GZ17-6.02 at different times and concentrations, and the proliferation assay was performed. GZ17-6.02 significantly inhibited the proliferation of these cells in a dose (1–100 μg/ml) and time-dependent (24–72 h) manner. After 72 h of treatment, the IC_50_ values of MiaPaCa-2 and S2-007 cells were determined as 8 μg/ml and 16 μg/ml respectively (Supplementary Figure 1A and 1B). Due to the differences in IC_50_ values, both cell lines were treated the cell's sub-lethal doses of IC_50_ for further experiments. Based on those observations the doses of 3 μg/ml or 6 μg/ml GZ17-6.02 for MiaPaCa-2 and 6 μg/ml or 12 μg/ml for S2-007 cells were used respectively for further experiments. Moreover, GZ17-6.02 did not affect the proliferation of HPNE up to 20 μg/ml treatment that indicated that GZ17-6.02 has a much lower effect on non-cancerous pancreatic ductal cells than cancer cells. To determine the long-term effect of GZ17-6.02 treatment, the cancer cells were incubated with the mentioned doses of the mixture for 72 h, and then the cells were allowed to grow in normal drug-free media. The colony formation in both the cell lines was significantly reduced suggesting that the drug is stable and has an irreversible effect on PDAC cells (Supplementary Figure 1C and 1D). To understand the effect on the movement of cancer cells, we then investigated the effect of GZ17-6.02 on cancer cell migration. Interestingly, we found that the compound has a significant effect on wound healing potential after 24 h of treatment (Supplementary Figures 1E and 1F). GZ17-6.02 also significantly inhibited the invasion of MiaPaCa-2 and S2-007 cells using Boyden chamber following 24h of treatment, suggesting that the compound has anti-invasive property (Supplementary Figure 1G and 1H). After confirming these we used the IC_50_ dose to see the mRNA level of the PDAC related genes and found that GZ17-6.02 reduces the expression of these genes (Figure 2A) along with the stem cell markers and SHH pathway (Figure 2B). Western blot analysis of stem cell markers (Figure 2C) and SHH pathway proteins (Figure 2D) confirmed a reduction in protein expression after GZ17-6.02 treatment. Pancosphere formation was also inhibited after treatment (Figure 2E) which was expected as the SHH pathway has been shown to play a role in the spheroid formation through stem cell differentiation mechanism. To understand the underlying mechanism of GZ17-06.02 mediated cell growth inhibition, cell cycle distribution was evaluated (Supplementary Figure 2). GZ17-6.02 significantly inhibited growth-related proteins pEGFR and pAKT in S2-007 cell lines (Supplementary Figure 2A). The compound significantly inhibited cell cycle progression as reflected by the significant reduction in G0/G1 phase after 72 h of treatment. Since there were fewer cells in the G0/G1 phase, we investigated cell cycle-related proteins by western blot (Supplementary Figure 2C). We found that there was significant downregulation of cyclin D, which plays a pivotal role in cell cycle progression in the G0/G1 phase. Cyclin A and cyclin B were also significantly down-regulated; however, cyclin E remained unchanged (Supplementary Figure 2C). To explore further the mechanism of cell death, apoptosis study was investigated using flow cytometry and western blot of Bax/Bcl2 expression (Supplementary Figure 3B). GZ17-6.02 treated S2-007 cells induced apoptosis as showed by the results of flow cytometry using Annexin V-FITC/PI (Supplementary Figure 3A and 3B). Significant numbers of apoptotic cells were found, particularly in early and late stages of apoptosis (Supplementary Figure 3B). The apoptotic proteins, Bax and Bcl2 significantly affected due to the treatment of GZ17-6.02 and caspase-3 is cleaved in the treated cells (Supplementary Figure 3C and 3D). Further, caspase 3/7 activity was verified to ensure apoptosis in the treated cells (Supplementary Figure 3E).

### H3K27ac could be targeted and reduced by GZ17-6.02 in PDAC cells thus reducing the transcription of the associated oncogenes

**Figure 3:**
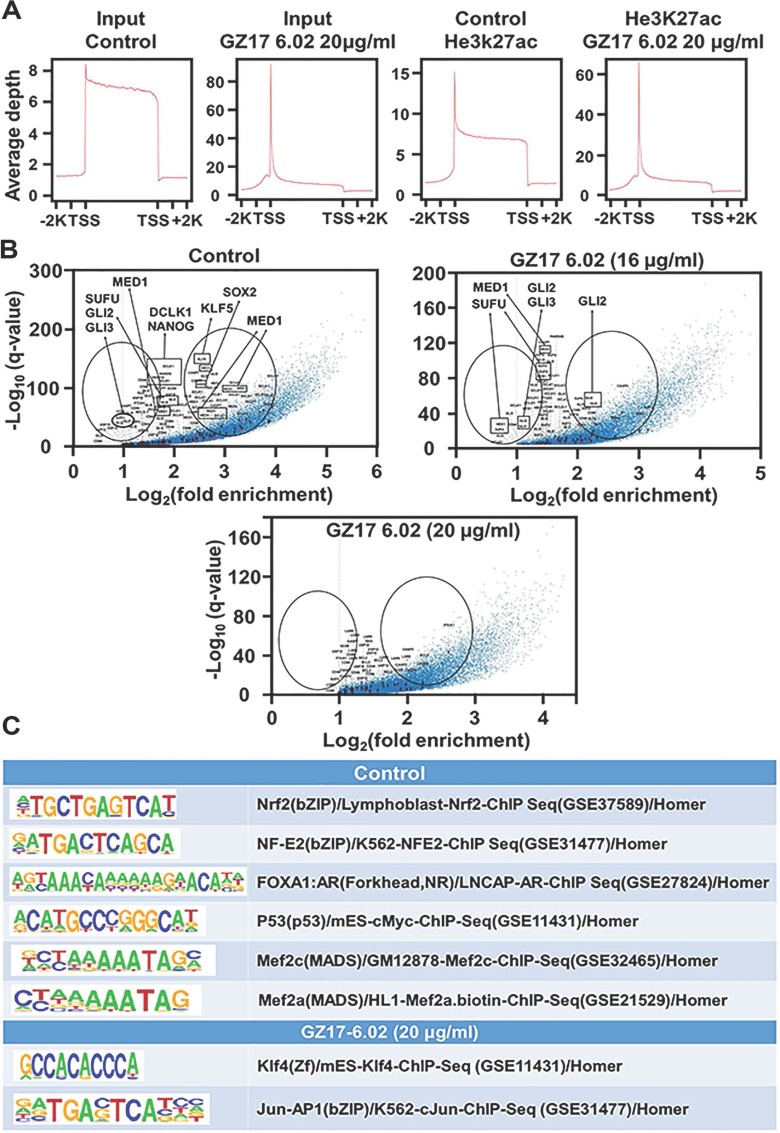
Effect on SEs following different doses of GZ17-6.02 (**A**) The treatment of S2-007 PDAC cells by GZ17-6.02 in higher doses (20 μg/ml) showing the TSS site of input using H3K27ac antibody, (**B**) whereas log curve showing SEs affected in higher doses. (**C**) The motif of KLF4 and JUN-AP1 were affected by the treatment.

To assess whether the H3K27 acetylation could be targeted leading to a reduction in the associated gene expressions we treated the S2-007 cells with 16 μg/ml and 20 μg/ml doses of GZ17-6.02 that are close to the IC_50_ value. Then we used ChIP-IT express kit from active motif to perform the ChIP-Seq with H3K27ac. We saw very high peaks of H3K27ac in TSS site of the genes in a genome-wide way (Figure 3A) verifying that this acetylation promotes transcription as those are found in abundance in the transcription start site. We checked how the enhancer landscape is altered after the drug treatment showing the change in acetylation after treatment and plotted the associated genes in a scatter plot in a negative log scale (Figure 3B). The data suggested a decrease in the H3K27 ac marks associated with the genes of the reported super enhancer components and the SHH pathway. This result suggests that GZ17-6.02 acts and has the potential to alter the associated gene expression epigenetically and affect the progression of cancer. We did the motif analysis to see the highest repeated motifs and elucidate whether there is any difference after the treatment. However, the motifs found to be repeated in all the groups were of NRF2, NF-E2, FOXA1, P53, MEF2C, and MEF2A showed no difference in control, and treated groups (Figure 3C). However, on the motifs for KLF4 and JUN-AP1 were repeated in the group treated with a higher dose (20 μg/ml).

### OCT-4 could be targeted and reduced by GZ17-6.02 in PDAC cells thus reducing the transcription of the oncogenes

**Figure 4:**
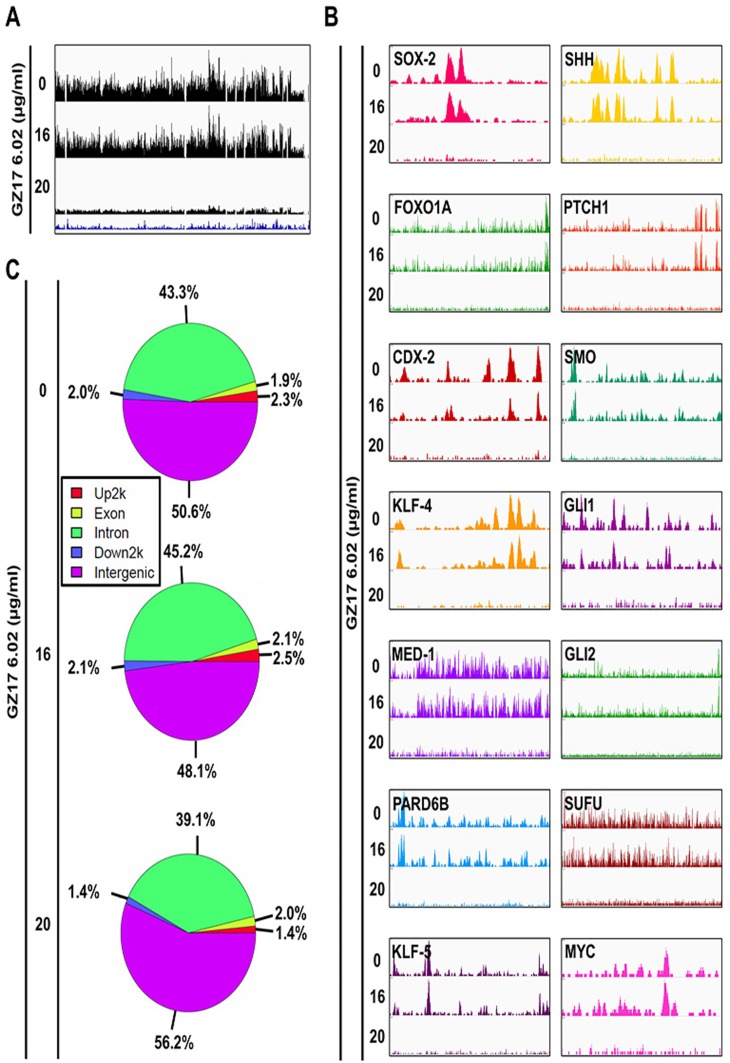
Detection of reduction of binding of OCT-4 (enhancer) at different sites associated with SEs following treatment with GZ17-6.02 (**A**) Occupancy of OCT-4 throughout the genome and the effect of GZ17-6.02 treatment on it. (**B**) OCT-4 signal (peaks) associated with the oncogenes, master transcription factors and SHH pathway indicating its occupancy at these regions that is significantly affected by GZ17-6.02 in different doses. (**C**) Pie chart of the treated groups compared to untreated groups.

As mentioned, co-occupancy of murine ESC genomic sites by master transcription factors is highly predictive of enhancer activity. Therefore, we sought to identify enhancers using ChIP-Seq dataset for OCT-4 (currently other master transcription factors are under investigation). When the enhancer landscape was evaluated to identify the alteration in control and treated group a significant reduction in acetylation level at TSS of OCT4 was observed in the 20 μg/ml treated group compared to untreated group unlike the H3K27ac global level (Figure 4A). OCT-4 binding was significantly altered and reduced to a great level for the observed Super-enhancer element genes, oncogenes, stem cell markers, and the SHH pathway. As seen in the mRNA levels from RNA-Seq data that OCT-4 transcription is reduced on drug treatment. Incoherence with the low expression data of OCT-4, our ChiP-seq data with OCT-4 further validated the significant decrease in the occupancy of OCT-4 across the entire genome and on cancer-specific genes. The lower expression of OCT-4 protein results in lower availability as a transcription factor, lowering the DNA-binding hence less ChIP product. The same is also represented in the peak digram (Figure 4B). However, while observing the pie chart of the treated groups compared to untreated groups, we did not find any significant changes in the regions, like H3K27ac, in this case, is also more than 90% of both *gain* and *loss* regions lie outside of the promoter (both downstream and upstream 2k) and correlate with the expression changes of nearby genes suggesting that they represent enhancer elements (Figure 4C). The data suggest a possible regulatory mechanism of GZ17-6.02 in regulating PDAC progression.

### GZ17-6.02 acts on the SHH pathway to reduce cancer stem cells

Multiple signaling pathways are known to be important for stemness, including the Wnt/β-catenin, Notch and SHH pathways [23]. To resolve the pathway by which GZ17-6.02 regulates the stem cell pathway, we did whole transcriptome shotgun sequencing (RNA-Seq). Interestingly, we observed that SHH/GLI pathway is significantly affected following treatment with GZ17-6.02 along with other proliferation and apoptotic markers. The heat-maps of hierarchically clustered genes show the relative differences in the expression between S2-007 control and treated cells (Figure 2B) respectively. Genes were mean centered on their normalized (fragments per kilobase per million sampled reads: FPKM) read counts for clustering. The hierarchical clustering was created with the ‘Euclidean’ distance as the distance matrix and the ‘average’ linkage as the linkage method. These results suggested us to investigate the SHH signaling pathways to assess the role of GZ17-6.02 on stemness. The SHH/GLI pathway is well known to play a significant role in developmental and cancer biology [24, 25]. SHH/GLI pathway is known to promote self-renewal of cancer stem cells (CSCs) by transcriptionally regulating expression of various genes including tumor metastasis [26, 27]. SHH signaling is initiated by the binding of SHH protein to its cognate receptor Patched (PTCH) on the cell surface [28]. This phenomenon impairs Smoothened (SMO) that hinders downstream signal transduction. To confirm the suppression of SHH signaling pathway by GZ17-6.02, we performed western blot analyses for proteins involved in the pathway including SHH and the downstream signaling regulators PTCH, SMO, SUFU, GLI1, and GLI2. Treatment with GZ17-6.02 (6 and 12 μg/ml) showed a significant effect in the expression of all these proteins (Figure 2D). SHH, SMO, SUFU, GLI1, and GLI2 were downregulated, whereas PTCH was upregulated. These data suggested that GZ17-6.02 affects PDAC stem cells in part through suppression of the SHH signaling pathway. The computerized molecular docking using Autodock Vina software, the binding of SHH with individual compounds of GZ17-6.02 (curcumin, harmine and isovanillin) identified where curcumin showed higher binding energy (−7.0 Kcal/mol) than harmine (−5.8 Kcal/mol) and isovanillin (−4.6 Kcal/mol) although their binding sites are different as sown in superimposed image (Supplementary Figure 4A and 4B). The individual compound did not affect proliferation after 72 h in both MiaPaCa-2 and S2-007 (Supplementary Figure 4C and 4D) but most interestingly, GZ17-6.02 did not affect proliferation in HPNE as much as in cancer cells, and the individual compounds did not show any difference at the doses used in GZ17-6.02 (Supplementary Figure 4E). To validate the inhibition of SHH pathway, we performed thermal shift assay (CETSA) to study the thermal stabilization upon GZ17-6.02 binding (Supplementary Figure 4F). We found that upon incubation with 12 μg/ml of GZ17-6.02 for 2h in S2-007 cells, the GZ17-6.02 binds with SHH protein and stabilizes the protein up to 58° C whereas in control unbound SHH denatured and precipitated in elevated temperature. Hence, GZ17-6.02 inhibited the SHH pathway by interacting with SHH. Next, cancer cells were incubated with GZ17-6.02 to predict, whether GZ17-6.02 has any binding with CSC marker proteins like DCLK1, EPCAM, LGR5, and SOX9, but we did not find any binding of GZ17-6.02 with CSCs (Supplementary Figure 8). These results suggest that GZ17-6.02 can bind to SHH and not its downstream proteins to inhibit cancer progression. Next, to validate the binding of SHH with GZ17-6.02, we performed series of experiments with the ‘SMO’ inhibitors- vismodegib (VIS) and cyclopamine (CYC) either alone or in combination with GZ17-6.02 on S2-007 and MiaPaCa-2 cells. We observed that IC_50_ value of VIS and CYC was 5 μM and 8 μM on MiaPaCa-2 whereas, 7 μM and 10 μM on S2-007 respectively (Supplementary Figure 5A and 5B) after 72 h of treatment. VIS was more potent to inhibit PDAC cells, so we chose VIS for combination study with GZ17-6.02 on S2-007. We found that the combination of GZ17-6.02 and VIS at their ½ IC_50_ dose significantly inhibited proliferation as compared to individual treatments following 72 h of treatments. Moreover, pre- or post-treatment (after 4 h intervals) with GZ17-6.02 and VIS showed a similar effect on proliferation following 72 h of treatment (Supplementary Figure 5B). Next, we observed that the combination of these two compounds significantly inhibited spheroid formation (Supplementary Figure 5C). Moreover, western blot analysis demonstrated that SMO was significantly inhibited by the treatment of VIS either alone or with a combination of GZ17-6.02, whereas SMO was not inhibited by the treatment of GZ17-6.02 alone. On the contrary, GZ17-6.02 significantly inhibited SHH either alone or in combination with VIS. GLI1 was significantly inhibited by both the compounds (Supplementary Figure 5D and 5E). It was suggested that GZ17-6.02 specifically inhibited SHH and not its downstream proteins to mitigate the SHH signaling pathway.

### Tumor growth and CSC markers could be reduced *in vivo* by GZ17-6.02

**Figure 5:**
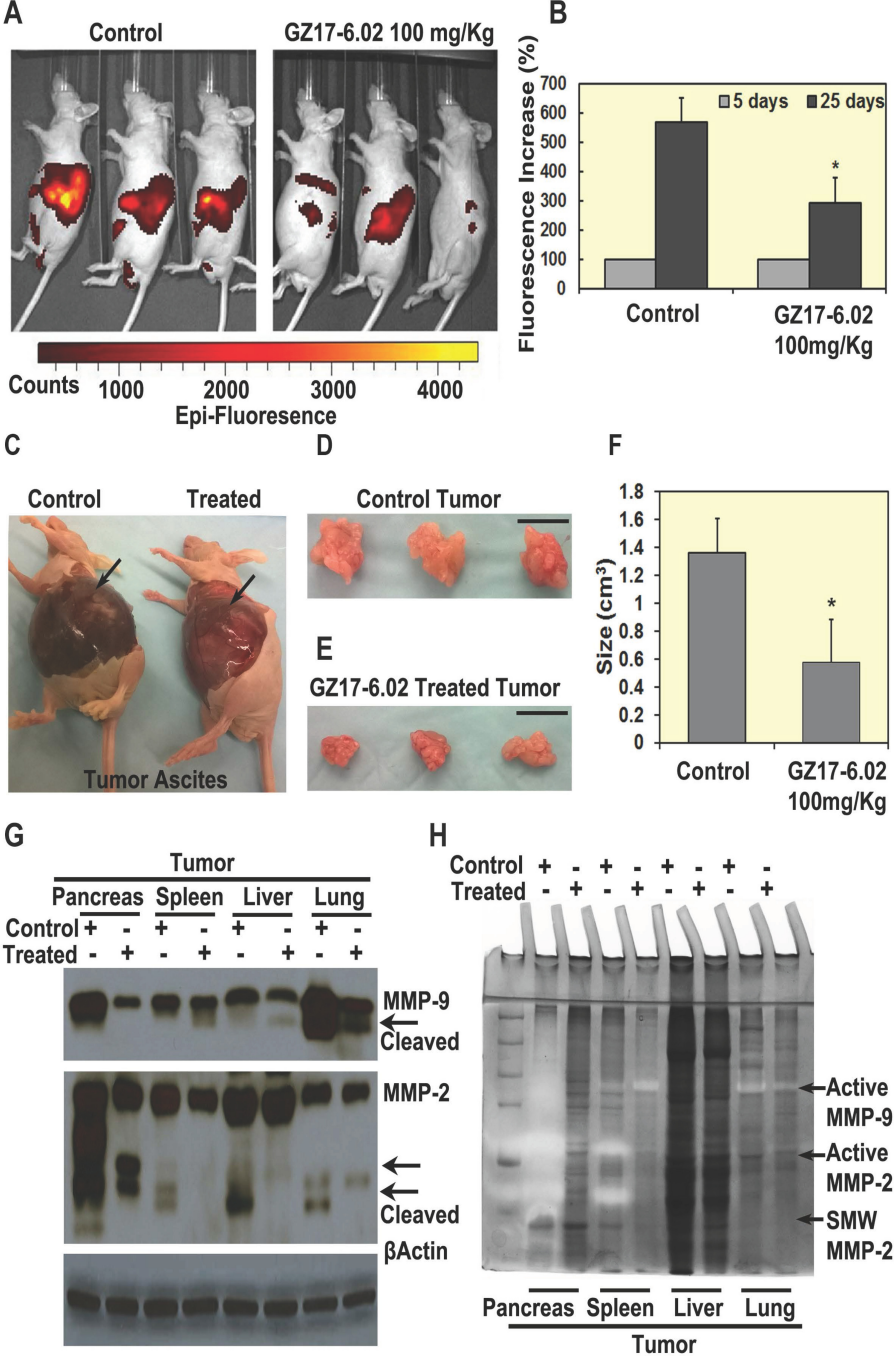
GZ17-6.02 inhibit tumor growth *in vivo* (**A** and **B**) Treatment of GZ17-6.02 (100 mg/kg) on orthotopic mouse tumor showing fluorescence, there were significant differences of Td-tomato fluorescence in tumors of control and treated mice (*N* = 6) after 20 days of treatment, (**C**) Tumor ascites fluid decreased in treated group with comparison to control (arrow), control mouse has larger ascites. (**D**, **E** and **F**) Size of tumors is significantly inhibited by the treatment in comparison with control or untreated animals. Bar = 1 cm. (**G** and **H**) Effect of GZ17-06.12 on metastatic markers Matrix metallopeptidase (MMP)-9 and MMP-2 in primary pancreatic tumor and metastatic spleen, liver and lung tissues of control and treated groups. (G) Western blots showing a decrease in expression of active MMP-9 and MMP-2 in the treated group than control, (H) Zymography, showing the clear bands, which depicts the reaction of active MMPs with their substrate gelatin showing by arrows.

To further investigate the effect of GZ17-6.02 on PDAC, we proposed the orthotopic tumor model in nude mice. For this study, we used S2-007 cells because these cells are characteristically more metastatic than other PDAC cells and demonstrated the development of tumors within a few weeks. Td-tomato transfected S2-007 cells were injected orthotopically in the pancreas of athymic nude mice. Tumors were allowed to develop for 5 days. Then, the mice were treated with GZ17-6.02 (100 mg/Kg/day), given by oral gavages for 20 days. After 5 and 20 days of treatment, tumor growth was monitored by a bioluminescence imaging system (IVIS). The tumors were monitored twice per week until the mice were euthanized after 25 days. Tumor incidence was 100% in all the animals with increased formation of ascitic fluid. However, the tumors in the GZ17-6.02 treated group was significantly reduced in comparison with the untreated controls (Figure 5A) with significant low fluorescence signal from tumors (Figure 5B and 5C). The size of tumor ranges from 1.1–1.7 cm^2^ in control and 0.3-0.8 cm^2^ in the treated group respectively (Figure 5D–5F). Furthermore, western blot analysis demonstrated that metastatic markers, MMP-9 and MMP-2 were significantly affected in primary pancreatic tumors and other metastatic organs (lung, liver, and spleen). Zymography elucidated the proteolytic activity of both MMP-9 and MMP-2 (both active and short molecular forms) in GZ17-6.02 treated mice as compared to control mice in the primary tumor and metastatic organs (Figure 5G and 5H). To determine whether the regression in tumor growth by GZ17-6.02 is due to inhibition of proliferation, apoptotic cell death or both, we first determined the expression of phosphorylation of EGFR and AKT. Western blot and immunohistochemistry analyses demonstrated significantly reduced levels of phosphorylated EGFR and AKT in the primary tumor and metastatic tissues upon treatment with GZ17-6.02 (Supplementary Figure 6A and Supplementary Figure 7E–7F). To establish the anti-proliferative effect of the drug, we performed western blot of proliferative markers in primary tumor and metastatic tissues. Phosphorylation of EGFR and AKT were significantly reduced in primary pancreatic tumors, spleen and liver tissues where metastasis was observed (Supplementary Figure 6E and 6F). Moreover, the SHH pathway (Supplementary Figure 6B and 6E) and CSC markers DCLK1, EPCAM, LGR5 and SOX9 (Supplementary Figure 6C and 6D) were significantly downregulated in GZ17-6.02 treated mice than control, depicting that GZ17-6.02 affects CSCs *in vivo* (Supplementary Figure 5C). Collectively, our data suggested that GZ17-6.02 significantly reduces tumorigenesis both in *in vitro* and *in vivo* PDAC mouse models.

## DISCUSSION

About 45,750 patients in the USA die of pancreatic cancer every year of which 85% of the total cases are referred as PDAC. Current therapeutic regimens have limited effectiveness; therefore, novel therapeutic approaches are needed to treat this disease. As we know targeted cancer therapies are drugs or other substances that block the growth and spread of cancer by interfering with specific molecules known as targets, that are involved in the cancer progression, growth and metastasis. However, finding effective agents with few serious side effects have been a major challenge. The aim of this study was to identify major specific targets in PDAC and target it with an effective substance that has the potential to reduce the progression of cancer both *in vitro* and *in vivo*.

Data analysis from bio-specimens is precarious to cancer research because they provide an extraordinary amount of biological information, translated in the language of cells, genes, and proteins that can identify the biological characteristics of cancer cells. Also, these databases survey a very large number of samples for each tumor type that ensures the statistical power needed to produce a comprehensive and wide-ranging genomic profile of each cancer type, which provides information for identifying the best targets for drug development. We started our ChIP-Seq by profiling H3K27ac because Heinz *et al*. showed by elaborate studies that out of all the marks available for the wide range of human samples the histone H3K27ac modification was superior to the others in that it identified a large fraction of OSN-Mediator SEs while minimizing excess sites [29, 30]. However, to perceive any one cell's gene expression program, co-occupancy of ESC genomic sites by the master transcription factors OCT-4, SOX-2, and NANOG is highly predictive of enhancer activity [30, 31]. Thus, ChIP-Seq with all the enhancers are to be followed this initial experiment. The first SE identified in mouse embryonic stem cells using ChIP-Seq and bioinformatics analysis and those SE clusters have master transcription factors such as OCT-4, SOX-2, and NANOG [13, 31]. ChIP-Seq data with H3K27ac was in accordance with the expression profile of the associated genes as found from the TCGA database.

The role of SHH signaling in PDAC is critical. Numerous studies have demonstrated that although activation of the SHH pathway is necessary for early embryonic specification of the gastrointestinal tract, downregulation of the SHH pathway is critical for pancreatic development. In adult pancreas, the activity of the SHH pathway is not limited and restricted to β-cells of the endocrine pancreas in the regulation of insulin production [32] but is also required for regeneration of the exocrine pancreas under circumstances such as injury or disease. Therefore, the aberrant activation of the SHH pathway in human PDAC was first reported by two independent studies. Later overexpression of SHH was observed in both pre-invasive and invasive epithelium in 70% of human PDAC samples, and detectable as early as PanIN1 and remains throughout all disease progression, but is absent in normal pancreas [33]. The anomalous expression of SHH is directly associated with oncogenic KRAS expression in PDAC. Ectopic expression of oncogenic KRAS^G12D^ in normal human pancreatic ductal cells lead to an increase of SHH transcript [34] indicating that SHH is a downstream effector of oncogenic KRAS^G12D^ in PDAC development. Therefore, overexpression of SHH was expected in our cell lines and is lower in normal cells, hence SHH could act as a more specific and potent target for cancer therapeutics. The compound GZ17-6.02 was effective in binding with SHH and downregulate the pathway.

To measure the different functional levels of the transcripts in a more detailed way, we analyzed RNA-Seq or whole transcriptome shotgun sequencing using untreated and treated PDAC cells with GZ17-6.02. The basal expression profiles of the treated and untreated cancer cells were compared. Bioinformatics analysis using heat-maps of hierarchically clustered genes showed the significant differences in the expression between control and treated cancer cells respectively. We found close to a two-fold decrease in expression of the super enhancer related elements after treatment. Moreover, DCLK1 and SHH signaling pathway demonstrated significant impairment due to the treatment of GZ17-6.02 in the cells. Based on this data, the SHH signaling pathway was next investigated. SHH signaling is one of the major molecules regulating CSCs in the progression of the tumor. SHH signals by binding to its transmembrane receptor, PTCH. In the absence of SHH ligands, PTCH associates and represses SMO [35]. When SHH binds to PTCH, SMO is released, triggering dissociation of transcription factors, GLI1 and SUFU, leading to transcription of an array of genes related to cell cycle, stemness, and metastasis [36]. Our results also indicated that GZ17-6.02 is a potent inhibitor of the SHH pathway, that in turn, inhibited self-renewal of cancer cells. After release from the complex, the transcription factor GLI translocate to the nucleus to bind to DNA in the promoter region and induces the expression of target genes like cyclin D1, cMYC etc. [37]. The significant down-regulation of GLI1 and GLI2 expression with its target genes cyclin D1 occurred simultaneously. In this study, we observed that GZ17-6.02 inhibits SHH, GLI1, GLI2 to suppress the downstream signaling. A growing body of evidence suggested that GLI activation could be modulated via EGFR/PI3Kinase/AKT signaling pathways because this can directly affect GLI expression [38, 39]. As EGFR signaling leads to a proliferation of cells, therefore, it can be predicted that inhibition of EGFR/AKT pathways by GZ17-6.02 leads to a noteworthy influence in SHH signaling and stemness of PDAC progression. We found a significant interaction between GZ17-6.02 and SHH using CETSA that involves thermal shifts of treated cells due to drug interaction with the target protein. Untreated samples' protein denatured and precipitated in a higher temperature compared to GZ17-6.02 treated samples due to its binding with SHH target protein. We also validated this interaction (GZ17-6.02 and SHH) using VIS, which competitively binds with SMO to inhibit the SHH pathway. We showed that GZ17-6.02 treatment on S2-007 has significantly inhibited SHH but moderately affected SMO. In summary, we can predict that GZ17-6.02 has significant binding with SHH to inhibit the signaling pathway.

**Figure 6:**
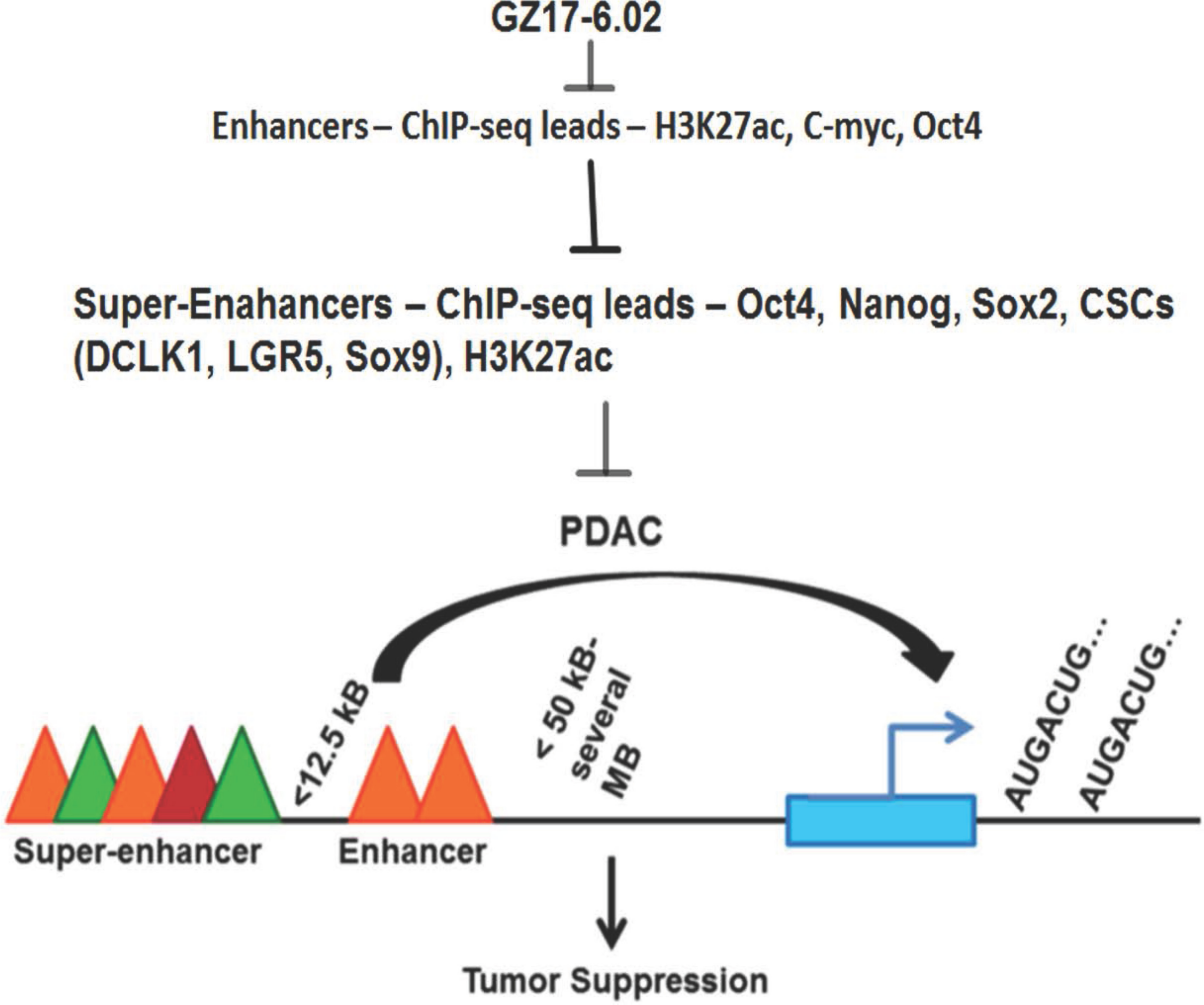
Schematic representation of effects of GZ17-6.02 inhibiting SE following tumorigenesis in PDAC

The goal of the study was to identify the active enhancer regions and whether they are specific for cancer as compared to non-cancer and then to target them with a drug which is more effective in cancer and has less effect on non-cancer cells with the idea to lower the side effects. We found decrease H3K27ac for the genes with hyperacetylated super enhancer regions affected by GZ17-6.02. We also found that another transcription factor OCT-4 is reduced leading to low expression and hence transcription activity of the other reported super-enhancer element in PDAC (Figure 6). As mentioned earlier in spite of low incidences PDAC is the fourth leading cause of cancer-related mortality because, by the time of diagnosis, more than 80% of cases are locally advanced or distally metastasized [40], and are not eligible for surgical resection, which is the most effective treatment option. Thus, the identification of mechanisms that lead to the progression of PDAC is the first milestone to be reached in cancer therapeutics. In this study, we first identified SE domains as a major contributor in PDAC progression and could be targeted by a potent mixture of natural compounds GZ17-6.02 because it has a significant effect on SE elements of cancer-promoting genes in PDAC.

## MATERIALS AND METHODS

### Cell culture and reagents

MiaPaCa-2 and Suit-2 (S2-007) were cultured in RPMI media along with 10% fetal bovine serum (Sigma Aldrich) and 1% antibiotics (Fisher Scientific) at 37° C in a humidified atmosphere containing 5% CO_2_. All the cell lines used in this study were within 20 passages after receipt or resuscitation. The cell lines were authenticated by Arizona State University.

### Chromatin immunoprecipitation (ChIP)

ChIP study was done using the ChIP-IT Express kit from Active Motif (cat no: 53008). First, intact cells were fixed using formaldehyde, which cross-linked and therefore preserved protein/DNA interactions. DNA was then sheared into small uniform fragments and the DNA/protein complexes were immunoprecipitated using H3K27ac antibody (Cat no: ab195415, Abcam). Following immunoprecipitation, the DNA was washed, cross-linking was reversed, and the proteins were removed by Proteinase K treatment. Eluted DNA was purified using Active Motif's Chromatin IP DNA Purification Kit (Cat no: 58002) then downstream analysis via qPCR or Next-generation sequencing was done.

### ChIP-Seq

For ChIP sequencing, samples were sent to BGI Americas Corporations. They used the BGISEQ-500 sequencing platform to perform the study. Standard bioinformatics analysis and production statistics were performed which includes read alignment, genome-wide distribution of ChIP-Sequencing reads, peak scanning, distribution peak related gene scanning, GO function analysis and difference analysis of multi-samples. Further bioinformatics analysis was performed.

### RNA-Seq

RNA was isolated from the cells using the RNeasy Mini kit from Qiagen (cat no: 74104). Then from the purified RNA Rapid Read RNA-Seq was prepared and bioinformatic analysis was performed.

### Proliferation assay

5 × 10^4^ cells /ml of MiaPaCa-2 and S2-007 cells were seeded in 96-well culture plates (Corning, USA). After 24 h, cells were treated for 72 h with various concentrations (5–200 μg/ml) of GZ17-6.02. Cell proliferation was determined by an enzymatic hexosaminidase assay as explained previously [41].

### Colony formation assay

5 × 10^2^ viable cells were plated in 6 well plates and allowed to grow for 24 h. The cells were then incubated in the presence or absence of GZ17-6.02 for 72 h. GZ17-6.02 containing medium was then removed, and the cells were washed in PBS and incubated for an additional 10 days in complete medium. The colonies obtained were washed with PBS and fixed in 10% formalin for 10 min at room temperature and then washed with phosphate buffer saline (PBS) followed by staining with Crystal violet. The colonies were counted and compared with untreated cells.

### Invasion assay

Invasion assay on MiaPaCa-2 and S2-007 cells with and without treatment of GZ17-6.02 was performed as described previously [42]. Briefly, 1 × 10^3^ cells were seeded in Boyden chambers, incubated for 24–48 h. The cells percolating the membrane were stained with DAPI followed by capturing of images.

### Cell cycle and apoptosis study

S2-007 cells treated with GZ17-6.02 for 72 h were trypsinized and suspended in PBS. Single-cell suspensions were fixed using pre-chilled 70% ethanol for 3 h and subsequently permeabilized with PBS containing 0.1% Triton X-100, 1 mg/ml propidium iodide (Sigma-Aldrich) and 2mg DNase-free RNase at room temperature. Flow cytometry was done with a FACSCalibur analyzer (Becton Dickinson, Mountain, View, CA, USA), capturing 10,000 events for each sample. Results were analyzed with ModFit LT TM software (Verity Software House, Topsham, ME, USA). Detection of apoptosis using Annexin V-FITC/PI dual staining was done by flow cytometry. Green FITC dye stains apoptotic cells whereas Propidium iodide stains necrotic cells with red fluorescence. After treatment, the cells were washed in cold PBS and resuspended in calcium-containing binding buffer (10 mM HEPES, 140 mM NaCl, 5 mM CaCl2; pH 7.4) at a concentration of 1×10^6^ cells/ml and stained for 15 min, with 5 μl Annexin V-FITC and 5 μl PI at 1 μg/ml (Cell signalling kit, USA). 10,000 cells were analyzed at an excitation wavelength of 488 nm and emission wavelengths of 530 nm for FITC fluorescence and 610 nm for PI fluorescence. The percentages of viable (Annexin V^−^PI^−^), early apoptotic (Annexin V+PI−), late apoptotic/necrotic (Annexin V+PI+) and necrotic cells (Annexin V-PI+) were evaluated with the CellQuestPro^®^ software (Becton, Dickinson, Heidelberg, Germany).

### Western blot analysis

Immunoblotting was performed as previously described [2]. Briefly, MiaPaCa-2 and S2-007 cells or tumors were homogenized, and the lysates were centrifuged at 18000 × g, for 1h at 4° C to precipitate the particulates. The supernatant was then collected and used for immunodetection of proteins. Antibodies were purchased from Cell Signaling Technology (Beverly, MA, USA), Santa Cruz Biotechnology Inc (Santa Cruz, CA, USA) and Abcam (Burlingame, CA, USA). Specific proteins were detected by the enhanced chemiluminescence system (GE Healthcare, Piscataway, NJ, USA).

### Pancosphere assay

Cells were cultured in RPMI 1640 supplemented with 20 ng/ml bFGF, 10 mL/500 mL of 50X B27 supplement, 20 ng/ml of EGF (all from Life Technologies) at low densities (3 × 10^3^ cells/mL) in 6 well low adhesion plates. Cells were treated with GZ17-6.02. After 5 days, the number and size of pancospheres were determined using Celigo (Cyntellect Inc., San Diego, CA, USA). For second and third passages, cells were grown in the absence of these compounds.

### Real-time reverse-transcription PCR

Total RNA was isolated from cells using Qiagen kit and reverse-transcribed with Superscript II reverse transcriptase in the presence of random hexanucleotide primers (Invitrogen). cDNAs were then used for real-time PCR using Jumpstart Taq-DNA polymerase (Sigma-Aldrich) and SYBR Green Nucleic Acid Stain (Molecular Probes). Crossing threshold values for individual genes were normalized to β-actin. Changes in mRNA expression were expressed as fold change relative to control. Primers used in this study were as follows: β-actin: 5′CTGATCCACATCTGCTGG-3′and 5′-ATCAT TGCTCCTCCTCAGCG-3; cyclin D1: 5′-AATGACC CCGCACGATTTC-3′ and 5′-TCAGGTTCAGGCCTT GCAC-3′.

### Cellular thermal shift assay (CETSA)

Established protocol for the assay has been described previously [43]. Briefly, S2-007 (70% confluent) cells were treated with GZ17-6.02 for 2 hours, harvested by scrapping then centrifuged and resuspended in PBS. Cells were equally distributed in 9 tubes for heat stability assay starting from 37° C to 65° C in a thermocycler. The proteins were extracted by freeze-thaw methods followed by western blot to detect the desired protein.

### Computerized binding activity determination

The X-ray crystal structure of SHH (PDB ID: 4C4M) was downloaded from the protein data bank. All the docking calculations were done with AutoDock Vina software to analyze curcumin, isovanillin and harmine interactions with the 3D-structure of SHH. Autodock Vina is a free molecular docking program for faster drug discovery and virtual screening of drug molecules. It also offers high performance and increased accuracy as compared to its previous versions. Autodock Vina software is developed in the Molecular Graphics Lab at The Scripps Research Institute (http://vina.scripps.edu/). It analyzes a rapid energy assessment through pre-calculated grids of affinity potentials and follows a variety of algorithms to determine suitable binding positions. The 3D-grid box is generated containing all active site residues and a grid center co-ordinate consisting of grid spacing 1.0 A^0^ and 60×60X60 point size. All docking calculations were performed using default parameters of the Autodock tools. Total Kollman and Gasteiger charges were added to the protein and the ligand prior to docking. Lamarckian GA was used to find the best conformations. Approximately 10 conformations for each compound were selected. Later, the most stable conformation for curcumin, isovanillin and harmine was selected based on the scoring function and the lowest binding energy and visualized with Pymol.

### Immunohistochemistry

Paraffin-embedded tissues were cut into 4 μm sections, deparaffinized and blocked with Avidin/Biotin for 30 min. The slides were incubated with primary antibodies for overnight at 4° C. After three washes with PBS, the slides were treated with a broad spectrum secondary antibody (Invitrogen) and HRP conjugate for one hour and then developed with DAB (Invitrogen). Finally, the slides were counterstained with hematoxylin. The slides were examined in Nikon Eclipse Ti microscope under a 20X objective lens.

### Animals

Six-eight weeks old athymic female outbreed nude mice (nu/nu) were obtained from Charles River Laboratories, (Wilmington, MA, USA) and were used for tumor development. All animals were maintained in a sterile environment having 12-hr light/12-hr dark cycle daily. All the mice were maintained according to the standard guidelines of American Association for the Accreditation of Laboratory Animal Care with the approval of the Institutional Animal Care and Use Committee of the KUMC.

### Orthotopic PDAC tumor model in athymic female nude mice

S2-007 cells (1.0 × 10^6^) cells were injected into the pancreas. Gently agitated matrigel containing cell suspensions (without clump) was injected directly into the head/neck region of the pancreas of athymic nude mice (3-5 weeks of age) using a 25-gauge needle. After 7 days, tumor growth was monitored by the bioluminescence imaging system. The luciferase substrate D-luciferin (150 mg/kg) was injected bi-weekly for tumor-bearing animals. Briefly, mice were anesthetized with ketamine/xylazine cocktail. The left abdominal/flank region of the mouse was shaved using clippers and the mouse was placed on its right side. The left side of the mouse was wiped from the base of the neck to the tail with 70% ethanol (v/v) and then rubbed with Chlorhexidine scrub. A 1cm incision was made in the left abdominal flank with sterile microscissors slightly medial to the splenic silhouette, and the pancreas was exteriorized using sterile forceps. The underlying muscle was grasped and lifted with forceps and an incision was made to enter the abdominal cavity without causing any injury to the underlying organs. Carefully a pair of blunt-nose forceps was used to gently grasp the tip of the pancreatic head and externalize the pancreas/spleen in a lateral direction, exposing the entire pancreatic body. Tumor cells (1.0 × 10^6^/0.05 ml) was then injected into the pancreatic head with a 30-gauge needle. The pancreas/spleen was returned to the abdomen. The muscle layer was closed with a 4-0 or 5-0 coated absorbable branded suture (coated VICRYL suture), and then the skin was closed with wound clips. Mice were allowed to recover on a warm water pad and the wound clips were removed within 7 days post-surgery. A successful injection is defined as no apparent spillage of the cell suspension into the peritoneal cavity and the presence of tissue bleb at the injection site that contains injected volume. All mice were sacrificed after 21 days following treatment and tumors were taken out and stored at −80° C.

### Luciferase expression vector and animal imaging

pc.DNA 3.1(+) Luc2-tdT expression vector derived from Addgene. The expression vector stably transfected (Lipofectamine 2000) into control and JMJD1a knock-down cells (S2-007 and MiaPaCa-2) and selected with Neomycin. The tdT.Luc2 expressing cells (1 × 10^6^) were mixed with Matrigel (BD Bioscience). The cells were orthotopically injected into the pancreas of nude mouse, wait for two weeks to develop a palpable tumor. After 2 weeks, D-Luciferin (150 μg/kg) was injected via i.p into the animals. The animals were imaged by the luminescent imager.

### The cancer genome atlas (TCGA)

The gene expression data of TCGA was analyzed by using GEPIA (Gene Expression Profiling Interactive Analysis) online [44].

### Statistical analysis

All values are expressed as the mean ± SEM. Data were analyzed using an unpaired 2-tailed *t*-test. A *P* value of < 0.05 was considered statistically significant.

## SUPPLEMENTARY MATERIALS FIGURES


