# Supplementary material for: Super-enhancers: novel target for pancreatic ductal adenocarcinoma
Source: Oncotarget. 2019 Feb 22;10(16):1554–71. doi: 10.18632/oncotarget.26704 (PMC6422180; doi:10.18632/oncotarget.26704)
Supplement: Supplementary file 1 [file oncotarget-10-1554-s001.pdf]

Super-enhancers: novel target for pancreatic ductal adenocarcinoma

SUPPLEMENTARY MATERIALS

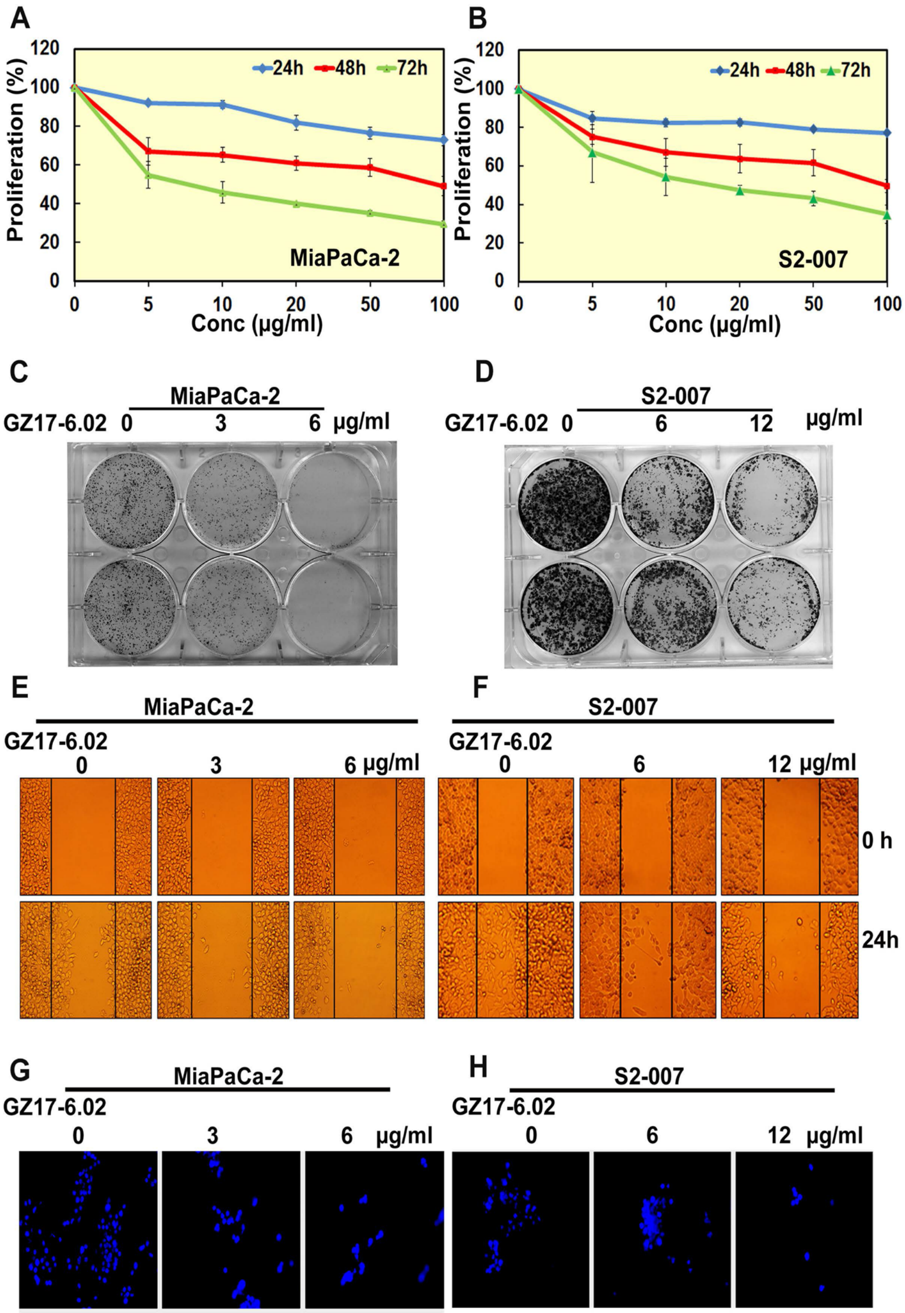

Supplementary Figure 1: Effect of GZ17-06.02 on proliferation, colony formation and migration in MiaPaCa-2 and S2-007 cells. (**A** and **B**) Showing hexosaminidase assay of proliferation. After 72 h of GZ17-6.02 treatment IC_{50} of MiaPaCa-2 was 8 μg/ml and IC_{50} of S2-007 was 16 μg/ml. (**C** and **D**) Colonies of both the cell lines were significantly reduced when treated with sub-lethal doses after 72 hours of treatment. (**E** and **F**) Migration of MiaPaCa-2 and S2-007 was markedly reduced when treated with GZ17-6.02 as seen after 24 hours of treatment. (**G** and **H**) Boyden Chamber Assays used to measure cell invasion. S2-007 and MiaPaCa-2 were significantly reduced upon GZ17-6.02 treatment after 24 hours of treatment. Cells were stained with DAPI and photographed under a fluorescence microscope.

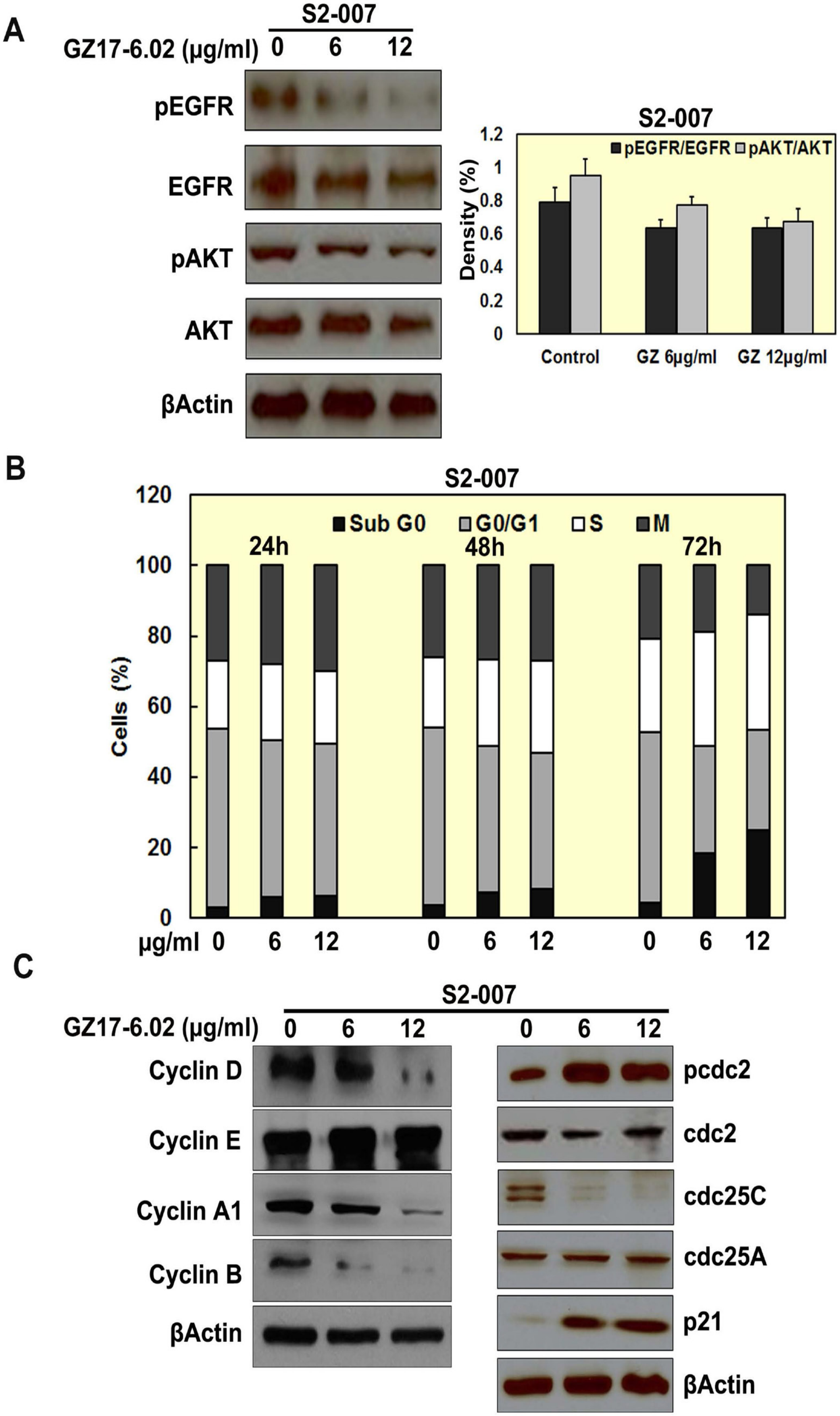

Supplementary Figure 2: Effect of GZ17-06.02 on growth regulatory and cell cycle proteins. (**A**) Western blots (WB) of pEGFR, EGFR, pAKT, AKT expressions showing the downregulation of pEGFR and pAKT upon treated with GZ17-6.02. Densitometry analysis was normalized with β-Actin, (**B**) Cell cycle analysis (bar diagram) with FACS showing a significant increase in sub-G0 phase and S phase after 72 h treatment with GZ17-06.02 in S2-007 cells. (**C**) WB of cell cycle-related proteins and p21 expression showing the disruption of cell cycle proteins when treated with GZ17-6.02 after 72 h.

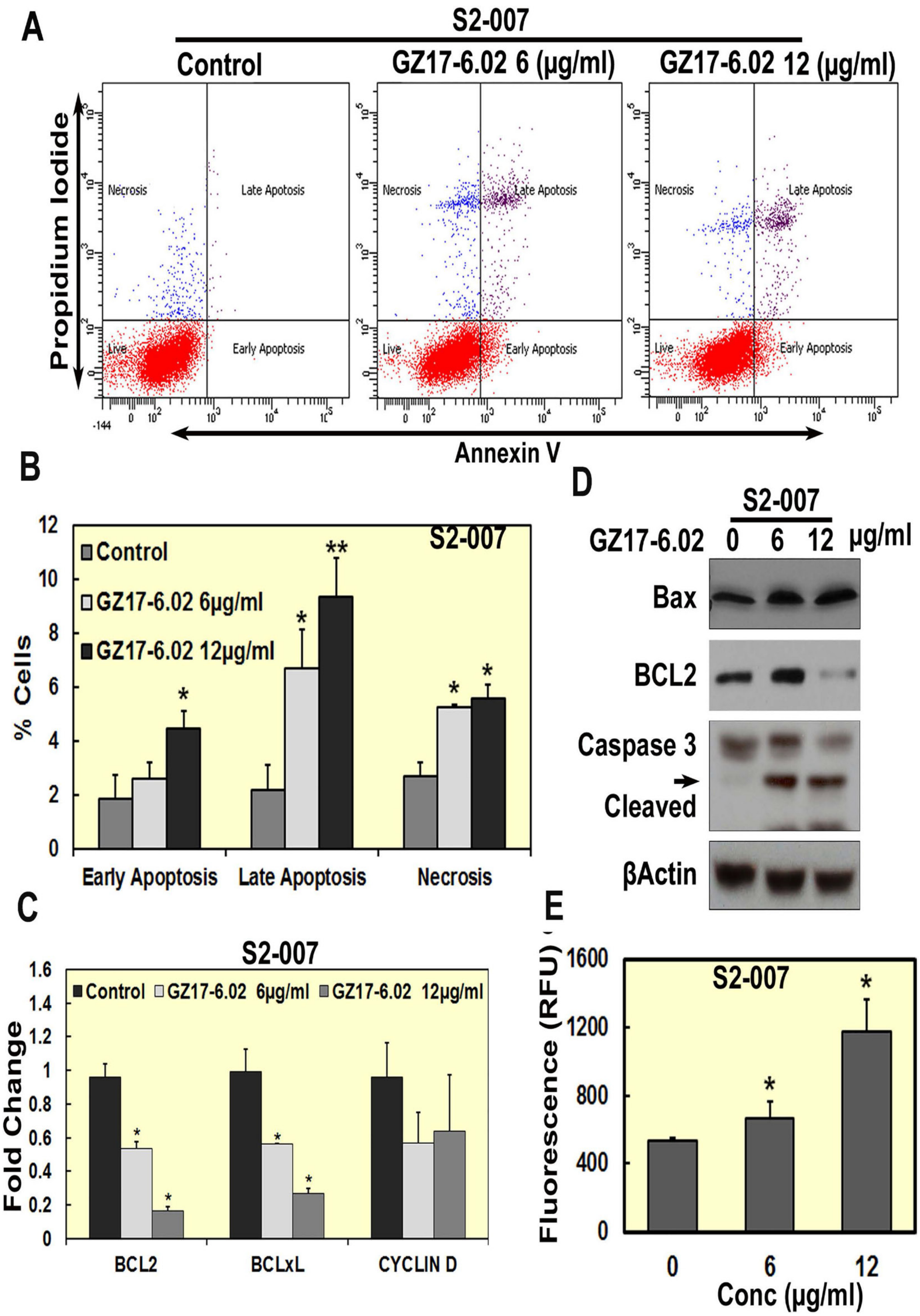

Supplementary Figure 3: GZ17-6.02 induces apoptosis. (**A** and **B**) FACS study of S2-007 cells after 72 h treatment of the compound showing significant apoptosis, (**C**) Real-time PCR of anti-apoptotic gene along with cyclin (**D**) GZ17-6.02 treated S2-007 cells significantly lowered in fold change in mRNA level of the anti-apoptotic gene. (D) Western blot showing apoptotic-related proteins. The bar represents the fold change of gene normalized to β-actin. (**E**) Significant increase in caspase 3/7 activity showing apoptosis in treated S2-007 cells after 72 h treatment of GZ17-6.02.

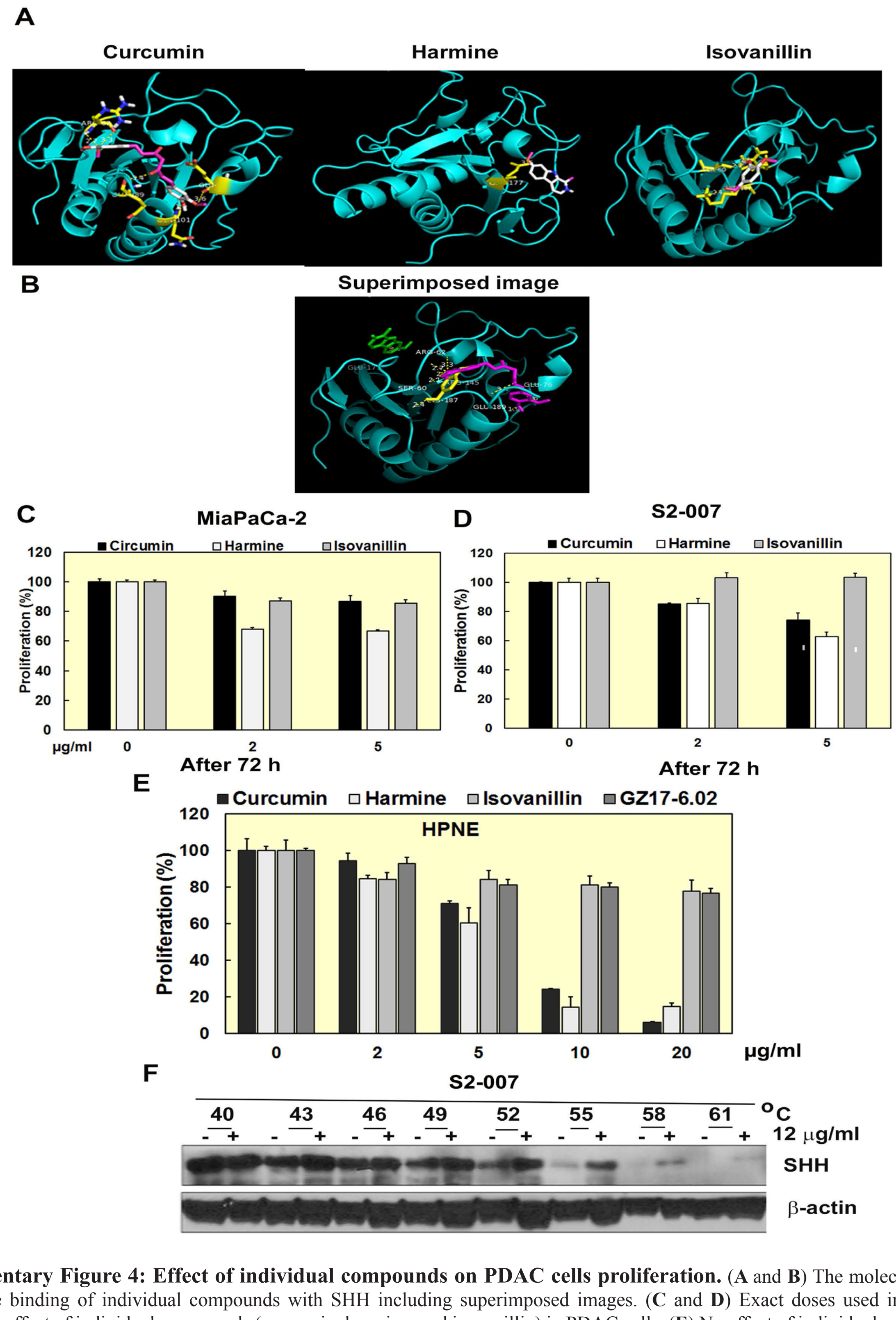

Supplementary Figure 4: Effect of individual compounds on PDAC cells proliferation. (**A** and **B**) The molecular docking showed the binding of individual compounds with SHH including superimposed images. (**C** and **D**) Exact doses used in GZ17-6.02 showing no effect of individual compounds (curcumin, harmine, and isovanillin) in PDAC cells. (**E**) No effect of individual compounds on non-cancerous cells. Higher doses (over 5 μg/ml) of individual compounds demonstrated some effect but not at the doses of GZ17-6.02. (**F**) CETSA assay showing GZ17-6.02 binding with SHH.

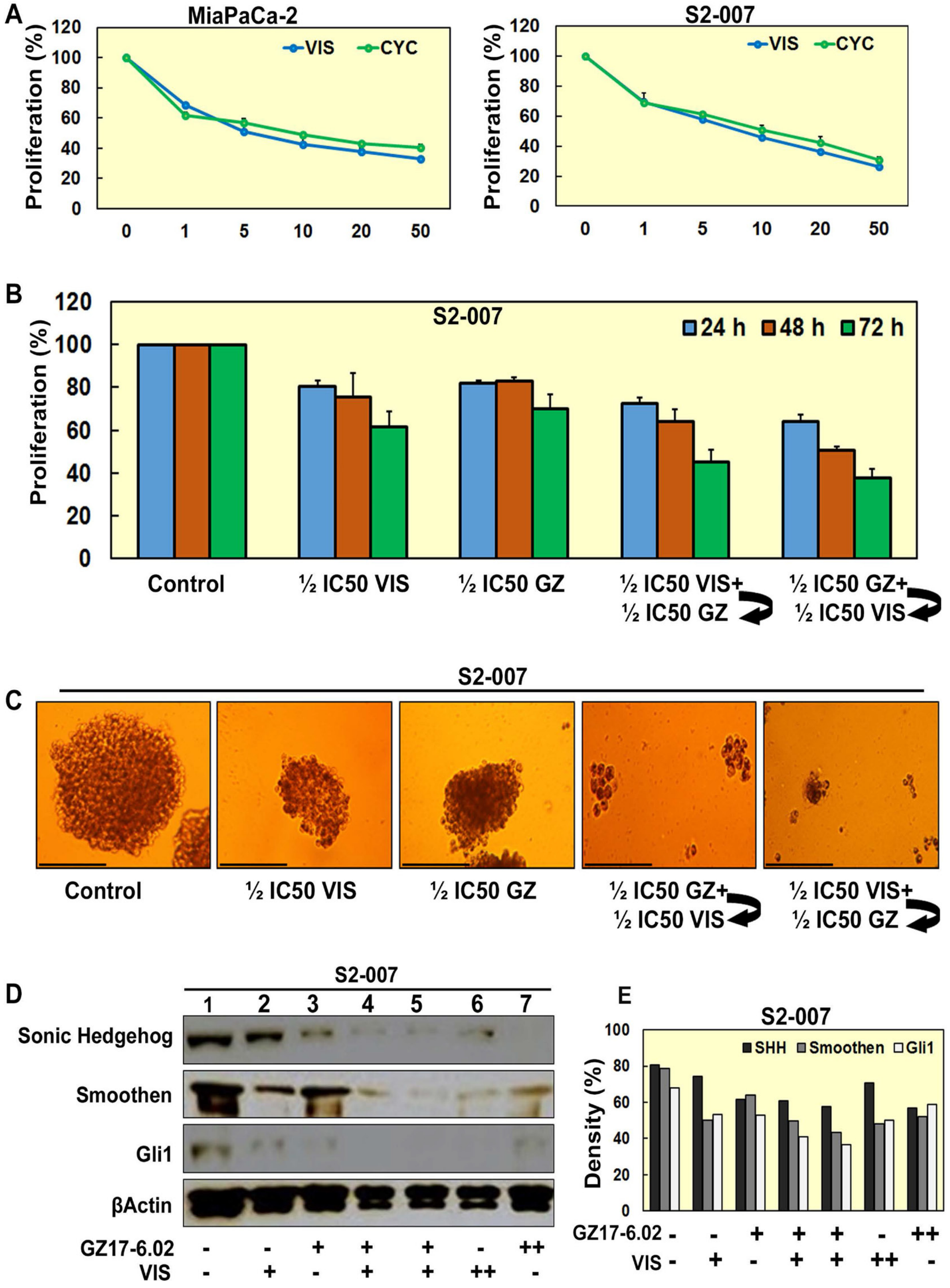

Supplementary Figure 5: Effect of Smo inhibitor VIS and CYC either alone or in combination in PDAC Cells. (**A**) Proliferation assay of VIS and CYC showed the IC_{50} of the compounds after 72 h of treatment. (**B**) Proliferation assay of 24, 48 and 72 h showing the effect of VIS and GZ17-6.02 in combination on S2-007. There were 4 h of latent between two drugs treated (black arrow). (**C**) Spheroid formation assay with the treatment of VIS and GZ17-6.02, black arrow showed the treated drug orientation. (**D** and **E**) Western blot showing inhibition of expression of SHH in a combination of VIS and GZ17-6.02 after 72 h of treatment. [+ = ½ IC_{50}; ++ = IC_{50}]. Lane 4 = VIS treatment followed by GZ17-6.02 (after 4 h). Lane 5 = GZ17-6.02 treatment followed by VIS.

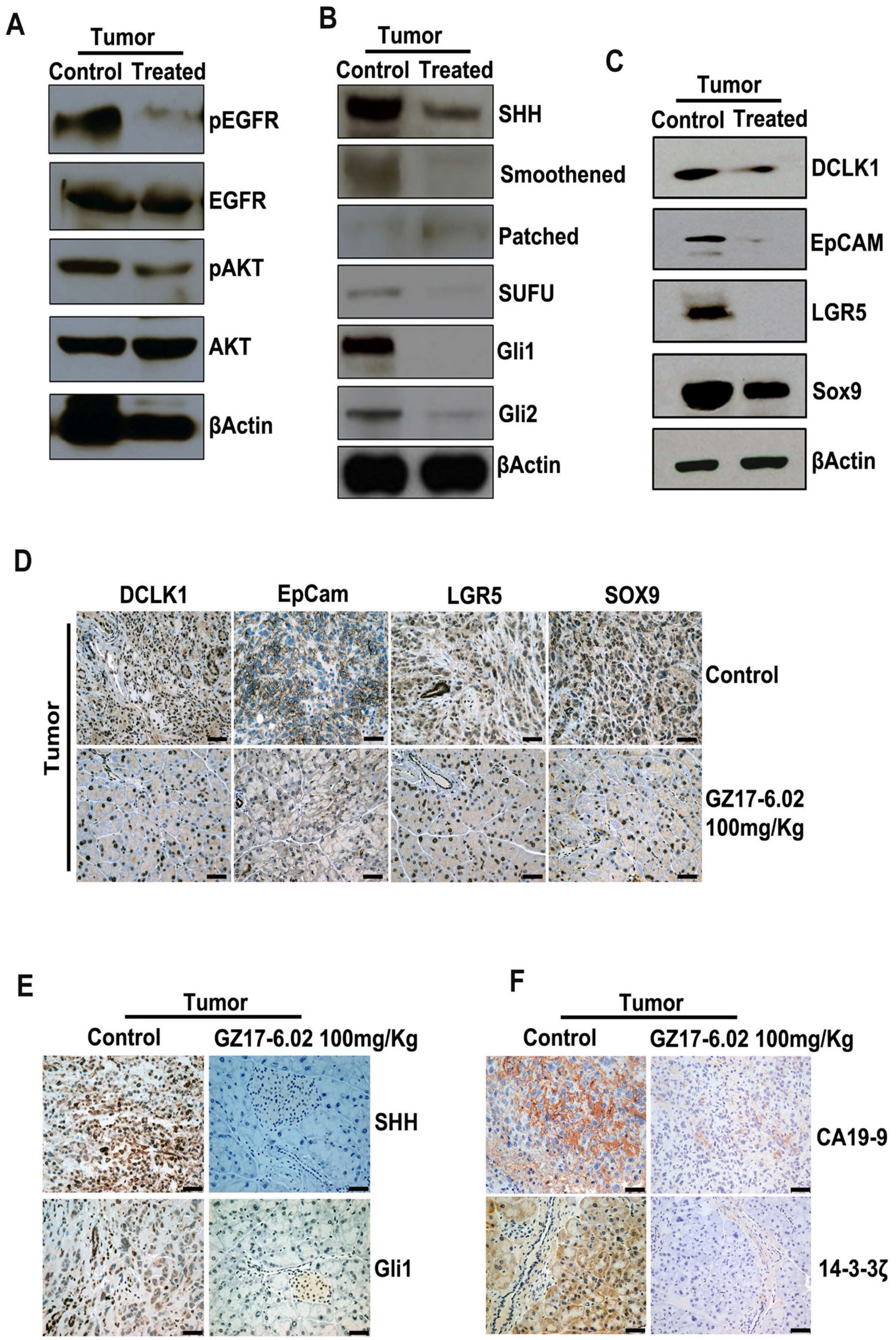

Supplementary Figure 6: (**A**) Effect of GZ17-6.02 on tumor proteins: pEGFR and pAKT of mice orthotopic tumors are affected by GZ17-6.02 treatment. (**B** and **E**) SHH pathway also inhibited in tumors following treatment. (**C** and **D**) GZ17-6.02 also inhibited CSC markers and both the markers for PDAC, CA119 and 14-33-e are affected by GZ17-6.02, (**F**).

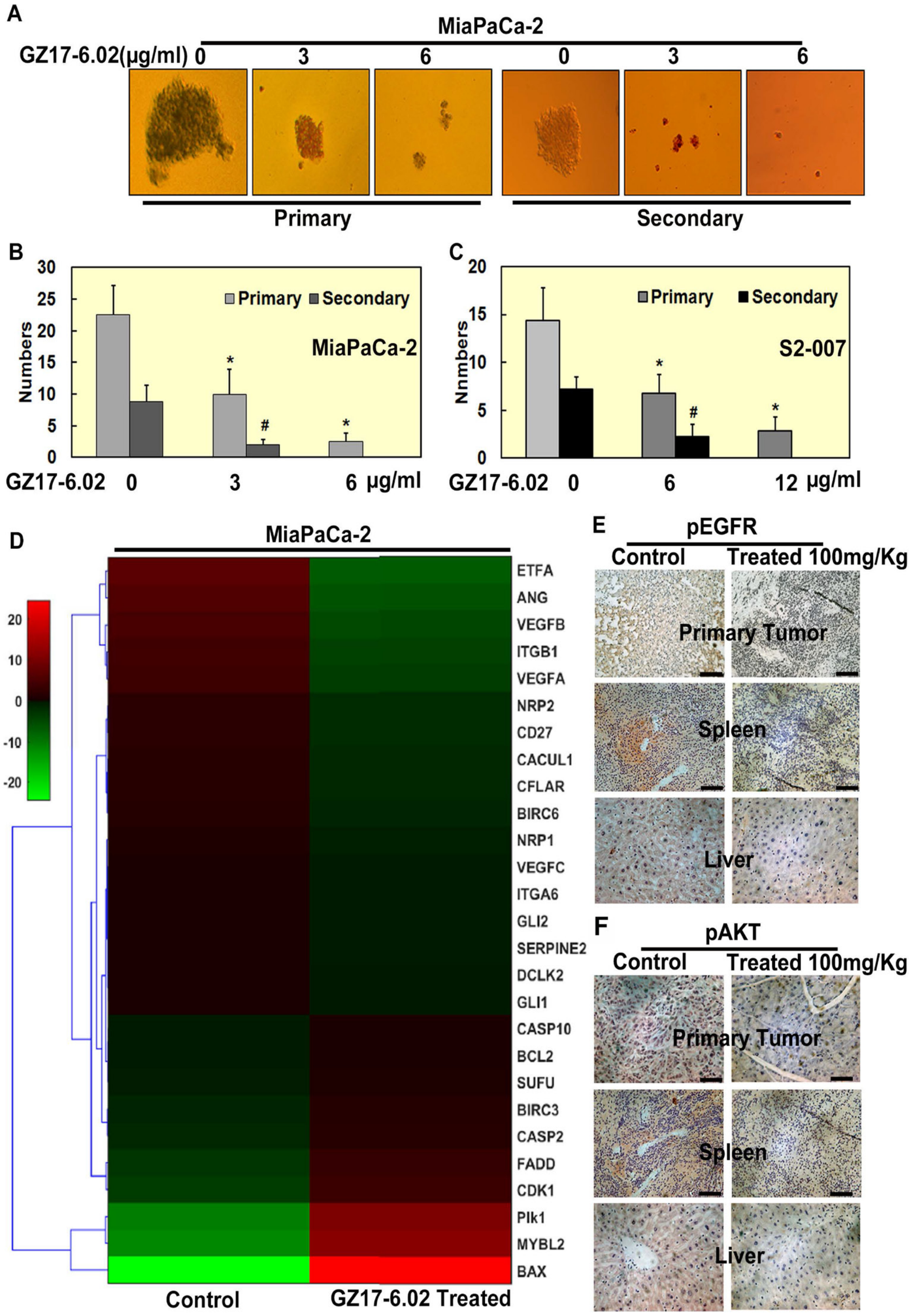

Supplementary Figure 7: (**A**–**C**) GZ17-6.02 inhibits cancer stem cells: Spheroid formation in S2-007 cells and the effect of GZ17-6.02 in the formation of primary and secondary spheroids. (**D**) Heat Map of RNA-Seq performed with control and treated MiaPaCa-2 cells showing the expression of a gene cluster in color difference, the color bar was showing on the left and dendrogram of the distance between the gene were shown with a blue line. (**E** and **F**) Immunohistochemistry of pEGFR and pAKT of primary tumors, spleen, and liver of orthotopic mice.

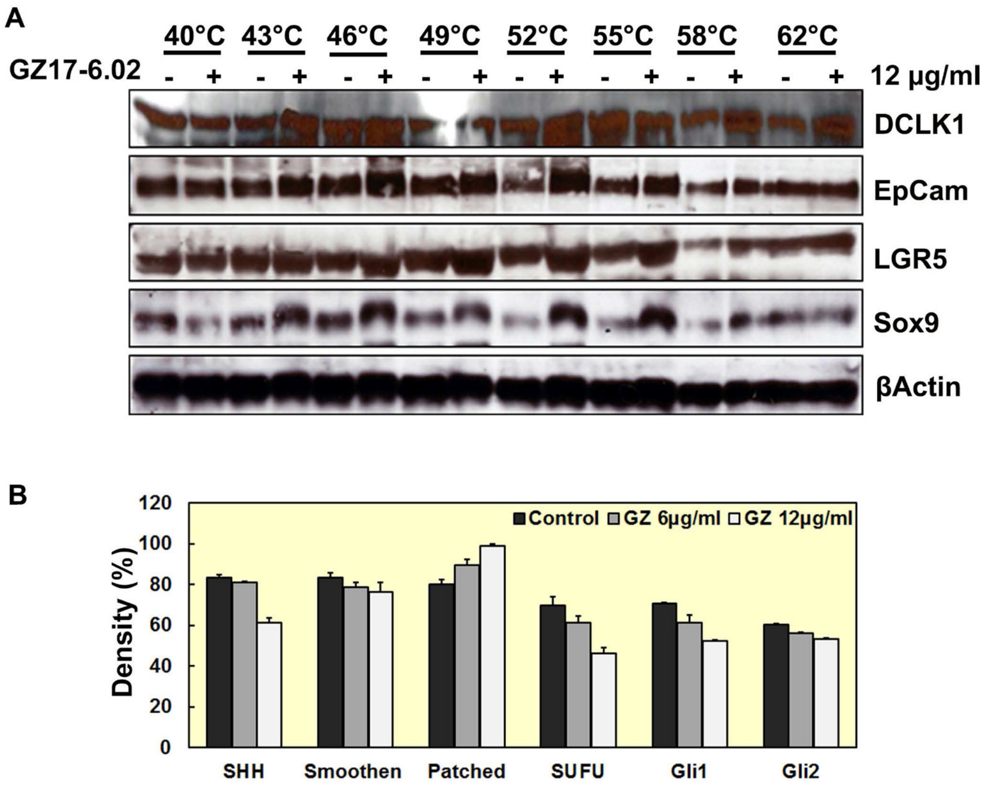

Supplementary Figure 8: (**A**) Negative Binding with other proteins with GZ17-6.02. The CSCs markers (DCLK1, EpCam, LGR5, and SOX9) were incubated with GZ17-6.02 to process CETSA followed by western blot showing no effect of binding with GZ17-6.02 to any of the CSCs. (**B**) Densitometry analysis of western blot of SHH pathway, expressions were normalized with the corresponding β-actin.
